# Determinants of Having Online Health Consultations During the COVID-19 Pandemic Among Middle-Aged and Older Adults in Germany: Representative Longitudinal Survey Study

**DOI:** 10.2196/60311

**Published:** 2025-05-26

**Authors:** Ariana Neumann, Hans-Helmut König, André Hajek

**Affiliations:** 1 Department of Health Economics and Health Services Research, University Medical Center Hamburg-Eppendorf, Martinistr. 52, Hamburg, 20246, Germany, 49 40 741054202, 49 40 741040261

**Keywords:** telemedicine, digital health, remote consultation, health services for the aged, patient acceptance of health care, COVID-19

## Abstract

**Background:**

During the COVID-19 pandemic, telemedicine services represented a widely implemented alternative to in-person doctor and therapist appointments. Consequently, rates of telemedicine use rapidly increased worldwide, also in Germany. Research regarding longitudinal determinants of telemedicine use is needed, particularly from nationally representative German samples, to improve understanding of the use behavior of major target groups such as middle-aged and older adults.

**Objective:**

This study aimed to longitudinally investigate determinants of online health consultation use among middle-aged and older individuals during the COVID-19 pandemic in Germany.

**Methods:**

Nationally representative longitudinal data of German middle-aged and older adults (≥46 years old) were taken from the German Ageing Survey (DEAS). Data from the Compact Survey (conducted between June and July 2020) and wave 7 (conducted between November 2020 and March 2021) of the DEAS were observed (pooled analytic sample N=5456). Having experienced consultations with doctors or therapists on online platforms served as the outcome measure. Associations with socioeconomic, health- and health behavior–related, psychological, and COVID-19–related determinants were tested using random effects logistic regressions.

**Results:**

In our sample, 49% (2673/5456) of participants were female and the mean age of the participants was 67.8 (SD 9.4) years. Past experience with online health consultations was reported by 10.3% (561/5456) of the sample. Online health consultation use was associated with high education (OR 1.43, 95% CI 1.06‐1.93; *P*=.02), poor self-rated health (OR 0.60, 95% CI 0.49‐0.75; *P*<.001), and higher frequency of physical activity (reference: low frequency; medium frequency: OR 1.58, 95% CI 1.15‐2.17; *P*=.005; high frequency: OR 1.73, 95% CI 1.09‐2.76; *P*=.02). Moreover, greater levels of loneliness (OR 1.43, 95% CI 1.06‐1.93; *P*=.04) and life satisfaction (OR 1.33, 95% CI 1.02‐1.73; *P*=.04) as well as perceiving the COVID-19 crisis as a greater personal threat (OR 1.08, 95% CI 1.01‐1.15; *P*=.02) were associated with having online health consultations during the COVID-19 pandemic.

**Conclusions:**

Online health consultation use does not seem to be exclusively associated with the health of middle-aged and older patients. Study findings emphasize the longitudinal association of education and psychosocial factors as well as health factors with telemedicine use during the COVID-19 pandemic in Germany. This knowledge may help to improve and adapt services to this patient group, which could contribute to higher utilization rates in the future. Future studies are needed to verify these initial findings under postpandemic circumstances and across different countries.

## Introduction

Worldwide, health care systems are facing multiple challenges in the future related to the delivery of services (eg, access and continuity of care), human resources (eg, staff distribution and sufficiency), as well as leadership and governance (eg, strategic policies) [1]. In particular, the increasing prevalence of noncommunicable diseases, disability, and multimorbidity in connection with population aging calls for new solutions in health care (eg, [2]). According to predictions of the World Health Organization (WHO) [3], the number of global deaths due to noncommunicable diseases may increase from 36 million (2008) up to 52 million in 2030.

Promising methods for the delivery of health care in the future include digital solutions such as telemedicine [4]. The WHO Global Observatory for eHealth [5] defines telemedicine as follows: (1) its purpose is to provide clinical support, (2) it is intended to overcome geographical barriers, connecting users who are not in the same physical location, (3) it involves the use of various types of information and communication technology, and (4) its goal is to improve health outcomes. Major strengths of telemedicine include improved access to care and information, time and cost savings, as well as convenience and flexibility [6,7]. Multiple studies evaluated the effectiveness and cost-effectiveness of telemedicine services. The services seem to be effective and can produce at least comparable effects to in-person services [8,9]. Moreover, the current literature suggests that telemedicine can be a cost-effective service delivery method [8-11]. In addition, providers as well as patients seem to be highly satisfied with the services [8,9,12].

Despite its benefits, telemedicine implementations were only limited and remained at a low level up until the occurrence of the COVID-19 pandemic [13-15]. To prevent further spreading of the virus and relieve the health care system, major changes in the delivery of health care services had to be made in response to the pandemic [15]. Telemedicine represented a valuable tool to avoid personal contact during health consultations. Consequently, the global telemedicine utilization increased tremendously [13-16]. For example, Koonin et al [17] reported a 154% increase in telehealth visits in the United States in March 2020 compared with March 2019. Also in Germany, which is the focus of our study, the proportion of contract physicians and psychotherapists who offered and billed for telemedicine services increased from 6.1% (2019) to 24.6% (2021) [18]. Particularly among the psychotherapeutic care sector, utilization rates were high in Germany [18]. Since many nonessential in-person medical appointments were canceled or postponed due to the pandemic in Germany [19,20], remote health consultations represented a valuable alternative format to assure the continuity of care in spite of pandemic circumstances. This digital transformation of the German health care system was facilitated by the introduction of laws including the Digital Health Care Act [21] and the Digital Health Application Ordinance [22], which supported the prescription of health care apps, provision of video consultations, and integration of digital provider networks. In the German health care system, health insurance is compulsory with about 90% of the population having statutory and 10% having private health insurance [23]. The implementation of the new laws enabled the coverage of digital health care by statutory insurances, ensuring that telemedicine users incur no additional costs.

Considering population aging, telemedicine is particularly relevant for the older patient groups. Telemedicine was found to be effective in older adults [24-26]. For instance, van den Berg et al [25] reviewed 68 interventional telemedical studies with a controlled design examining older adults and found that none of the included studies reported better outcomes for the control group (eg, randomized or matched control groups that received usual in-person care). Moreover, older adults seem to accept and are satisfied with the services [24,27]. Taking older adults’ higher need for health care and probable mobility restrictions into account, older adults can particularly benefit from the remote format. Nevertheless, older patients use telemedicine services less often than younger age groups and report multiple barriers to using the services (eg, [28-32]). For example, Wilson et al [30] identified factors such as physical difficulties (eg, visual or hearing impairments), privacy concerns, lack of experience, training, or support as barriers to the use of eHealth by older adults.

In Germany, older patients also used telemedicine services less frequently than younger patients did during the pandemic [18,33-36]. However, increasing telemedicine use in older age groups can be helpful for the future delivery of health care services in Germany to deal with problems such as physician shortages or increased demand for (long-term) care caused by demographic change [37]. Therefore, it is important to examine factors that are associated with telemedicine use in older adults to increase utilization in Germany. Previous international reviews highlighted older patients’ characteristics associated with higher telemedicine use [38-40]. This included younger age, higher education, higher self-efficacy, greater experience or skills in using electronic devices, access to technology and internet, greater social support or influence, higher (health-related) motivation, and greater openness to experience as well as fewer privacy concerns and less severe health impairments.

So far, only limited evidence from Germany regarding the determinants of telemedicine use during the pandemic exists. The previous studies stressed the association of socioeconomic (male or female sex, younger age, high or low education, social status, living in an urban area, and having children under 18 years), psychosocial (loneliness, digital literacy), health (mental or physical health problems), and COVID-19–related factors (higher perceived severity of COVID-19 infection, having had COVID-19 infection, subjective COVID-19–related challenges, COVID-19–related cognitive preoccupation, anxiety, and worries) with telemedicine use [18,34-36,41-43]. Very few studies exclusively looked at middle-aged and older adults in Germany during the pandemic. These studies highlighted the positive association of telemedicine use with education, living with a partner in the same household, mental (ie, anxiety and depression) or physical health problems, loneliness, life satisfaction as well as forgoing medical treatment due to the fear of being infected by the coronavirus [44,45]. A large variety of determinants was observed in the existing studies and more research is needed to further explore and verify the findings.

In addition to the small number of studies that observe determinants of telemedicine use in middle-aged and older adults during the pandemic in Germany, hardly any studies examined the determinants longitudinally. Solely, von der Groeben et al [35] used a quasi-longitudinal design (ie, they observed cross-sectional data from 3 different time points) to detect determinants of patient use and attitude toward using video and telephone conferences in a population-representative sample of adults (18‐69 y) affected by depression during 3 different pandemic time points in Germany. Since telemedicine use and acceptance varied over the course of the pandemic (eg, [31,35,46]), it may be beneficial to consider more than just one time point when observing telemedicine use behavior during the pandemic. Moreover, the longitudinal approach gives further insight into the directionality of the relationships. Therefore, our study aimed to longitudinally investigate determinants of online health consultation use in a large representative sample of middle-aged and older individuals during the COVID-19 pandemic in Germany.

Expanding the knowledge regarding determinants of online health consultation use in middle-aged and older adults could help to identify target groups for telemedicine services, as well as groups that would benefit from additional support for using the services. Furthermore, this knowledge may help to adapt telemedicine services to the needs and preferences of middle-aged and older adults. Consequently, important practical and theoretical implications may be derived from our findings, which could foster greater use of telemedicine services among middle-aged and older individuals, ultimately helping to deal with future health care challenges posed by population aging.

## Methods

### Sample

Nationally representative cross-sectional and longitudinal data were taken from the German Ageing Survey (DEAS [47]). The DEAS focuses on the German middle-aged and older population (starting at 40 y) and aims to describe living conditions and diversity among this population as well as aging and social change processes that are related to this life stage [47]. The first wave of the DEAS was conducted in 1996, followed by further waves in 2002, 2008, 2011, 2014, 2017, 2020/2021. The survey has a cohort-sequential design in which new baseline samples were added in 2002, 2008, and 2014. The baseline samples of the DEAS were disproportionally stratified into age groups, gender, and region [47]. In response to the COVID-19 pandemic, an additional Compact Survey to measure the pandemic impact on middle- and older adults’ lives in Germany was implemented in 2020.

For the purpose of our study, data from the Compact Survey [48] and wave 7 [49] of the DEAS were observed. The Compact Survey was conducted from June until July 2020 and consisted of paper-and-pencil questionnaires that were sent to individuals who had taken part in the DEAS at least once in the past. The response rate for the Compact Survey was 57% (4823/8533) [50]. Due to the ongoing pandemic during wave 7, the usual computer-assisted personal interviews were replaced by telephone-administered interviews as well as an additional paper-and-pencil questionnaire. The data collection for wave 7 took place from November 2020 until March 2021 and the response rate was 66% (5402/8207) [51]. Overall, 4103 individuals participated in both surveys [51]. Since the last DEAS baseline sample was added in 2014, participants of the Compact Survey and wave 7 of the DEAS were at least 46 years old.

The DEAS is funded by the German Federal Ministry for Family Affairs, Senior Citizens, Women, and Youth and was conducted and developed by the German Centre of Gerontology. The fieldwork was carried out by the INFAS Institute for Applied Social Sciences.

### Ethical Considerations

Written informed consent was obtained from all individuals who participated in the DEAS, and opting out of the survey was possible at all times [47]. DEAS participants received an incentive (eg, €10 in DEAS wave 7; conversion rate US $1=€0.951 in 2022 [51]). Due to data protection guidelines, a data distribution contract needs to be signed prior to using the anonymized DEAS data [47]. The DEAS study complies with the Declaration of Helsinki and did not require further ethical examination since the criteria for the need of ethical approval were not met for this survey (eg, missing information regarding the study or aim of the study, examination of vulnerable groups or patients, high risk or burden for participants due to participation).

### Dependent Variable

For the sake of our study, we exclusively included DEAS participants, who indicated having access to the internet (Compact survey: 3858/4676, 83%; wave 7: 3676/4276, 86%). In both DEAS waves, participants were asked “How often do you use the Internet for the following purposes?.” Among the listed items was “Consultations with doctors and therapists via an online platform” in the Compact survey and “Providing consultations with doctors or therapists on online platforms” in wave 7 of the DEAS. The response format consisted of a 6-point Likert scale indicating use frequency as “never,” “less often or seldom,” “1 to 3 times a month,” “once a week,” “several times a week,” or “daily.” Since only a small number of participants had multiple consultations with doctors or therapists on online platforms, the outcome was dichotomized for our analysis (0=“never”; 1=“less often or seldom,” “1 to 3 times a month,” “once a week,” “several times a week,” or “daily”).

### Independent Variables

Based on theoretical considerations and previous research [38-40,44,45], different groups of determinants were considered. This included socioeconomic, health- and health behavior–related, psychological, and COVID-19–related determinants. Regarding socioeconomic characteristics, we examined sex, age, educational level (International Standard Classification of Education 97 [52]: low, medium, or high education), employment status (used, retired, and other or unemployed), household income, migration background (no, yes), area lived in (metropolitan districts, urban districts, [partially] densely populated rural districts, sparsely populated rural districts), residential form of partnership (no partner, partner in the same household, and partner not in the same household), and presence of children (no, yes).

Health-related factors included self-rated health (ranging from 0=very bad to 4=very good) as well as the frequency of physical activity and walks (ranging from 0=never to 5=daily) as measures of health-related behavior. For the sake of our analysis, the values for the frequency of physical activity and walks were divided into tertiles and were included as categorical variables (low, medium, and high frequency).

Psychological determinants that were considered were depressive symptoms, loneliness, attitude toward own aging, and life satisfaction. Depressive symptoms were measured using a 10-item German short form of the Center for Epidemiologic Studies - Depression scale [53] (CES-D; scores ranging from 0 to 30, higher values indicate more severe depressive symptoms) in the Compact survey. In wave 7 of the DEAS, depressive symptoms were measured using the German version of the 15-item CES-D [54] (scores ranging from 0 to 45, higher values indicate more severe depressive symptoms). The values for both scales were standardized to assure comparability between both surveys in our analysis. Both of these well-established instruments were evaluated in the past and have good psychometric properties [53,55,56]. Cronbach α values for both scales were 0.83 (Compact survey) and 0.84 (wave 7) and McDonald Omega was 0.85 (Compact survey) and 0.86 (wave 7) in our sample. In addition, loneliness was measured with the 6-item De Jong Gierveld Loneliness Scale [57] (scores ranging from 1 to 4, higher values indicate higher levels of loneliness). The scale has favorable psychometric properties [57] (Compact survey: Cronbach α=0.78, McDonald’s Omega =0.79; wave 7: Cronbach α=0.80, McDonald Omega =0.81). The self-perception of one’s own aging was examined using the German version of the 5-item Attitude Toward Own Aging subscale of the Philadelphia Geriatric Center Morale Scale [58,59] (scores ranging from 1 to 4, higher values indicate a more positive perception of own aging). This widely used scale was evaluated in different age groups in the past (eg, [60]). In our sample, Cronbach α was 0.77 (Compact Survey and wave 7) and McDonald Omega was 0.77 (Compact Survey and wave 7). Life satisfaction was measured with the German version of the Satisfaction with Life Scale [61,62] (scores ranging from 1 to 5, higher values indicate greater life satisfaction). The German version of the scale was evaluated in the past and showed good psychometric properties [63]. For this scale, the Cronbach α was 0.86 (Compact survey) and 0.84 (wave 7) and the McDonald Omega was 0.87 (Compact survey) and 0.86 (wave 7) in our sample.

Finally, we controlled for COVID-19–related determinants, which included perceiving the Corona crisis as a personal threat (scores ranging from 1=not at all a threat for me to 10=extreme threat for me), past infection with the Coronavirus by oneself (no, yes, and unknown), or by people from one’s personal environment (no, yes, and unknown) as well as the feeling of being able to influence the infection with the Coronavirus (scores ranging from 1=not at all to 7=entirely).

### Statistical Analysis

In the first step, sample characteristics of our pooled analytic sample were computed. The analytic sample consisted of individuals who participated in at least one of the 2 surveys (5456 observations corresponding to 3222 individuals). Second, random effects logistic regressions were calculated to test the associations of the determinants with online health consultation use. The random effects regression model considers the panel structure of the data and allows the inclusion of not only time-varying but also time-constant predictors in our model, under the assumption that unobserved unit-specific heterogeneity is not correlated with the independent variables [64]. When this independence assumption is fulfilled, the random effects model may be more efficient than the fixed effects model as it considers both between and within variation [64]. Our choice was supported by the Hausman test. The null hypothesis of the Hausman test states that both models (fixed and random effects model) are consistent while the random effects model is more efficient [64,65]. Therefore, the random effects model is preferred when the null hypothesis cannot be rejected. Since the Hausman test statistic was nonsignificant for our sample (*P*=.72), we used random effects models for our analysis. Stata (version 16.0, StataCorp) was used for the statistical analyses and the random effects logistic regression was calculated using the “xtlogit” command with the “re” option. The sample was stratified by sex and age groups (≤64 and ≥65 years) in additional analyses. Statistical significance was defined as an alpha level of *P*<.05. Missing data were handled using listwise deletion.

## Results

### Sample Characteristics

The pooled analytic sample characteristics for all included variables are presented in Table 1. In the pooled sample of the Compact survey and wave 7 of the DEAS, 49% (2673/5456) were female and the mean age of the participants was 67.8 (SD 9.4) years. When examining past consultations with doctors or therapists on online platforms, 10.3% (561/5456) reported past experience with online consultations.

**Table 1.** Analytic pooled sample characteristics (N=5456).

Characteristics^a^		Values
Consultations with doctors or therapists on online platforms, n (%)		
No		4895 (89.7)
Yes		561 (10.3)
Sex, n (%)		
Male		2783 (51)
Female		2673 (49)
Age (years), mean (SD)		67.8 (9.4)
Educational level, n (%)		
Low (ISCED^b^ 0‐2) or medium (ISCED 3‐4)		2455 (45)
High (ISCED 5‐6)		3001 (55.0)
Employment status, n (%)		
Employed		1745 (32.0)
Retired		3475 (63.7)
Other or unemployed		236 (4.3)
Monthly household income (€), mean (SD)^c^		4051.2 (11,806.1)
Migration background, n (%)		
No		5223 (95.7)
Yes		233 (4.3)
Area lived in, n (%)		
Metropolitan districts		1524 (27.9)
Urban districts		2014 (36.9)
(Partially) densely populated rural districts		1135 (20.8)
Sparsely populated rural districts		783 (14.4)
Residential form of partnership, n (%)		
No partner		1069 (19.6)
Partner in the same household		4152 (76.1)
Partner not in the same household		235 (4.3)
Having children, n (%)		
None		602 (11)
One or more		4854 (89)
Self-rated health, mean (SD)^d^		2.6 (0.8)
Frequency of physical activity, n (%)		
Low frequency		1589 (29.1)
Medium frequency		3234 (59.3)
High frequency		633 (11.6)
Frequency of walks, n (%)		
Low frequency		2135 (39.1)
Medium frequency		2147 (39.4)
High frequency		1174 (21.5)
Depressive symptoms, mean (SD)^e^		−0.1 (0.9)
Loneliness, mean (SD)^f^		1.8 (0.5)
Life satisfaction, mean (SD)^g^		3.9 (0.7)
Attitude toward own aging, mean (SD)^h^		3.0 (0.5)
Perceiving the Corona crisis as a personal threat, mean (SD)^i^		4.3 (2.1)
Oneself infected with the Coronavirus, n (%)		
No		5244 (96.1)
Yes		57 (1)
Unknown		155 (2.8)
People from personal environment infected with the Coronavirus, n (%)		
No		4517 (82.8)
Yes		853 (15.6)
Unknown		86 (1.6)
Feeling that one can influence the infection with the Coronavirus, mean (SD)^j^		4.7 (1.4)

a Due to differences in measurement tools, values for depressive symptoms had to be standardized. The standardized values should be interpreted as number of SDs by which the original values lay above or below their mean. For example, a value of 1 or -1 indicates that the reported overall score in the CES-D lays one SD above/below the mean CES-D score.

b ISCED: International Standard Classification of Education.

c Range (0-500,000); the conversion rate of USD to Euro was US $1=€0.846 in 2021 and US $1=€0.951 in 2022.

d Range 0-4. Higher values indicate better self-rated health.

e Range for standardized values –1.8 to 5.9. Higher values indicate more depressive symptoms.

f Range 1-4. Higher values indicate higher loneliness levels.

g Range 1-5. Higher values indicate greater life satisfaction.

h Range 1-4. Higher values indicate a more positive perception of own aging.

i Range 1=not at all a threat for me to 10=extreme threat for me.

j Range 1=not at all to 7=entirely.

### Regression Analysis

**Figure 1.**
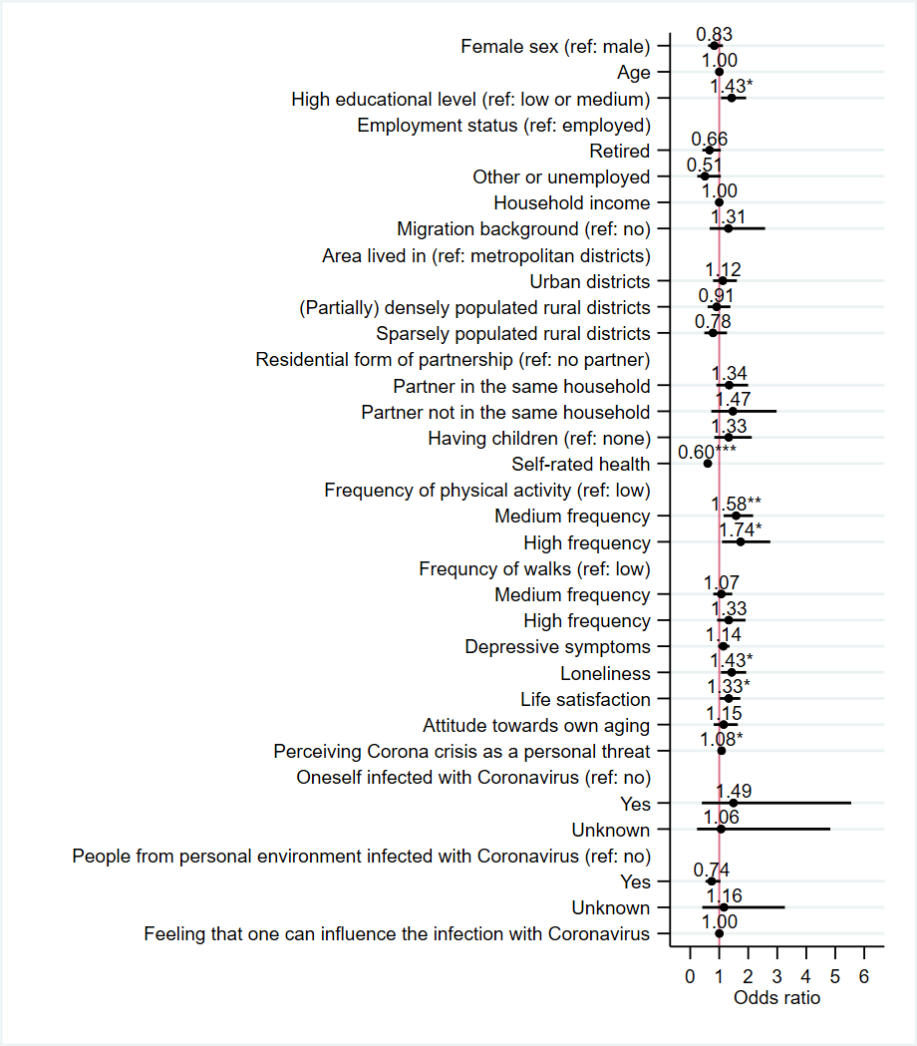
Results of random effects logistic regression for determinants of online health consultation use during the COVID-19 pandemic (N=5456). Odds ratios with 95% CI are reported. Unless stated otherwise, the reference category is always zero or absence of the characteristic. ref: reference category; *** *P*<.001; ** *P*<.01; * *P*<.05.

The results of the random effects logistic regression are presented in Figure 1 (Table S1 in Multimedia Appendix 1). The majority of the determinants were not significantly associated with online health consultation use during the COVID-19 pandemic. Nevertheless, we found a significant longitudinal association of the outcome with high education (OR 1.43, 95% CI 1.06‐1.93; *P*=.02), poor self-rated health (OR 0.60, 95% CI 0.49‐0.75; *P*<.001), higher frequency of physical activity (medium frequency: OR 1.58, 95% CI 1.15‐2.17; *P*=.005; high frequency: OR 1.73, 95% CI 1.09‐2.76; *P*=.02), higher loneliness (OR 1.43, 95% CI 1.06‐1.93; *P*=.04), greater life satisfaction (OR 1.33, 95% CI 1.02‐1.73; *P*=.04), and perceiving the Corona crisis as a greater personal threat (OR 1.08, 95% CI 1.01‐1.15; *P*=.02).

In additional analyses, we stratified the sample by sex and age group (Tables S2 and S3 in Multimedia Appendix 1 for more details). While online health consultation use in female participants was only associated with poor self-rated health (OR 0.62, 95% CI 0.45‐0.85; *P*=.004), in male individuals the outcome was associated with poor self-rated health (OR 0.59, 95% CI 0.44‐0.79; *P*<.001) as well as high education (OR 1.70, 95% CI 1.12‐2.56; *P*=.01), living with a partner in the same household (OR 2.27, 95% CI 1.13‐4.59; *P*=.02), living with a partner not in the same household (OR 2.86, 95% CI 1.04‐7.87; *P*=.04), higher loneliness (OR 1.94, 95% CI 1.25‐2.99; *P*=.003), higher frequency of physical activity (medium frequency: OR 1.88, 95% CI 1.22‐2.90; *P*=.004; high frequency: OR 2.17, 95% CI 1.17‐4.03; *P*=.01) and walks (high frequency: OR 1.99, 95% CI 1.24‐3.20; *P*=.004). In participants aged ≤64 years, online health consultation use was associated with poor self-rated health (OR 0.59, 95% CI 0.42‐0.83; *P*=.003) and higher frequency of physical activity (medium frequency: OR 1.91, 95% CI 1.14‐3.21; *P*=.01). In older participants (≥65 years), poor self-rated health (OR 0.61, 95% CI 0.46‐0.81; *P*=.001) as well as older age (OR 1.03, 95% CI 1.00‐1.07; *P*=.05), high education (OR 1.58, 95% CI 1.06‐2.37; *P*=.026), and higher loneliness (OR 1.52, 95% CI 1.01‐2.29; *P*=.04) were associated with online health consultation use.

## Discussion

### Principal Findings

Nationally representative longitudinal data from Germany were used to observe the longitudinal association of various determinants with the use of online consultations with doctors or therapists during the COVID-19 pandemic in a large sample of middle-aged and older individuals with access to the internet. Random effects logistic regressions revealed associations of education, health, and psychosocial factors with online health consultation use during the pandemic. In additional analyses stratified by sex and age, self-rated health was negatively associated with the outcome in all groups. Additional relationships with age, education, relationship status, and loneliness were only observed among the male and older (≥65 years) subgroups. Considering the limited evidence regarding determinants of telemedicine use (particularly based on longitudinal data), our longitudinal study considerably extend current knowledge on socioeconomic, health- and health behavior–related, psychological, and COVID-19-related determinants.

### Relation to Previous Research

In contrast to findings from other German samples [18,33,35,36,41,42,66], most of the socioeconomic determinants, such as sex or age, were not associated with online health consultation use in our sample. Nevertheless, a study that exclusively observed an older German sample also found no associations with socioeconomic characteristics [45]. This could imply that socioeconomic characteristics are less relevant for telemedicine use in middle-aged and older patients in Germany. However, the individual’s educational level was positively associated with the outcome in our analysis. This is in line with findings from other large German [33,34,36,42] and international samples [38,40,67,68] and could suggest that high education is linked to higher digital literacy as well as access to necessary technical equipment for using telemedicine. A systematic review by Estrela et al [69] examined 36 international articles on digital health literacy and highlighted its positive relationship with education. Correspondingly, in a large US sample of old-aged Medicare beneficiaries (≥65 years), Choi et al [70] found associations of younger age and higher income with telemedicine use during the COVID-19 pandemic, which disappeared when controling for technology-enabling factors (eg, information and communication technology device ownership or use experience). Consequently, technology-enabling factors could be particularly considered when trying to enhance telemedicine use among older patients. For instance, implementing supporting material (eg, leaflets or video instructions), technical support (eg, via telephone) or hybrid care combinations in clinical practice may be helpful to strengthen competence and digital health literacy in older patients.

Health and health behavior were associated with online health consultation use in our sample. Individuals who reported a poorer health status were more likely to use online health consultations during the pandemic. Likewise, severe health limitations or poor health were associated with telemedicine use in the German Survey of Health, Ageing and Retirement in Europe (SHARE) sample of middle-aged and older adults [45] and in international samples [68,71]. This association could have been caused by greater health needs in unhealthy individuals or may be connected to precautions due to the COVID-19 pandemic. Likewise, perceiving the Corona crisis as a greater personal threat was associated with online health consultation use. Telemedicine might have presented a treatment option for patients who were scared of becoming infected with the Coronavirus and wanted to avoid personal contact (eg, [72]). Fear of the Coronavirus and pandemic-related challenges were also associated with telemedicine use in other German samples during the pandemic [36,41,42,45]. Other COVID-19–related factors such as the infection of oneself or close others with the virus were not associated with the outcome in our sample. A reason for that could be that only a small proportion of our pooled sample was infected with the virus (1%) or knew someone in their close personal environment who was infected (15.6%). Furthermore, the frequency of physical activity was positively associated with online consultation use. Physical activity is an important determinant of health and was associated with higher utilization of preventive or office-based health services and lower use of inpatient or emergency care among adults in previous international research (eg, [73,74]). Therefore, physically active individuals seem to show greater levels of health awareness, which might have been connected to the higher telemedicine use in this group.

Some psychological factors were associated with the outcome in our sample. Whereas depressive symptoms and the attitude towards one’s own aging did not show an association, psychosocial factors including loneliness and life satisfaction had a significant relationship with online health consultation use. In the German SHARE sample, a positive association of depressive symptoms as well as loneliness with telemedicine use in middle-aged and older individuals was observed [45]. The mixed evidence concerning the relationship of depressive symptoms with telemedicine utilization might be explained by the different pandemic periods that were considered in the studies (Summer and Winter 2020 vs Summer 2021). In fact, depressive symptoms [75] and telemedicine acceptance [35] were found to have increased over the course of the pandemic. Regarding loneliness, Robbins et al [76] also observed higher loneliness rates among telemedicine users (telephone contacts), while in-person visits were associated with fewer feelings of loneliness among older adults (≥65 years) residing in the United States during the pandemic. It might be the case that older adults who indicated higher levels of loneliness were more open to using telemedicine services to satisfy their unmet social needs during pandemic times. Telemedicine services may be more accessible for older individuals (eg, no traveling for mobility-restricted individuals needed), which might encourage lonely individuals to take the initiative to foster social interaction through telemedicine appointments. Regarding life satisfaction, König et al [77] recently observed a positive correlation between life satisfaction and digital health literacy in a nationally representative survey of the population in Germany, which could explain the observed relationship between higher life satisfaction and online health consultation use in our sample. In addition, higher life satisfaction was associated with health-promoting behaviors in previous studies [78-80], which could have contributed to higher online health consultation use.

Additional analyses stratified by sex and age further highlighted the relationships with the determinants, especially in male and older participants. While online consultation use in females was only associated with health needs, male individual’s use behavior was additionally associated with psychosocial factors (ie, relationship status and loneliness), education, and health behavior (ie, frequency of walks and physical activity). The decision of male patients to use telemedicine services seems to be connected to additional factors and therefore more complex compared with female patients. Male patients may be facing additional barriers to telemedicine utilization. Therefore, future research is needed to explore gender-specific determinants and barriers to the use of telemedicine. Furthermore, particularly among individuals aged 65 years and older, loneliness, education, and age were associated with online health consultation use. Consequently, compared with individuals aged 40 to 64 years, older patients might be especially affected by disparities in education or loneliness.

When considering the international context, multiple reviews mainly based on quantitative cross-sectional, randomized controlled or qualitative studies observed determinants of telemedicine use in older age groups and found positive associations with educational level, health needs, and health-related motivation to use the services [38-40], which is in line with our findings. Moreover, they observed additional relationships (eg, with age, sex, and social support or influence), which we did not find in our analysis. Age or sex were not associated with overall telemedicine use in our sample. The reviews [38-40] mostly included studies from the United States or Europe. Telemedicine regulations differ substantially between Germany and the United States, but also in the European context. Whereas Germany has implemented national telemedicine coverage rules during the pandemic, large state-specific variation in insurance coverage and regulations exist in the United States [71,81], which may have caused additional barriers for older telemedicine users. Nevertheless, our stratified analyses indicated differences in determinants of use in the different sex and age groups. Furthermore, we observed a positive relationship of loneliness with telemedicine use, which is in contrast to the observed negative relationship of social isolation or lack of social support with telemedicine use in international studies [39]. Raja et al [82] reviewed studies of older adults in European countries and found that social support and lack of social support were both associated with using new technologies, including telemedicine. Social support may be crucial when older adults are faced with problems when learning new technologies, which was also observed in US samples [68]. Nevertheless, lack of social support or loneliness might also motivate older adults to try out new technologies to address their social needs, which we observed in our sample. Moreover, the discrepancies might also be explained by differences in regulations or access to telemedicine care (eg, variations in out-of-pocket payments, supply, or complexity of use). For instance, the requirement for additional out-of-pocket payments or high barriers to use may lower the probability of using telemedicine services for the primary purpose of social interaction. Future research concerning differences in psychosocial determinants across different countries is needed.

### Strengths and Limitations

The nationally representative, large DEAS sample of middle-aged and older individuals in Germany represents a key strength of our study. Middle-aged and older adults are a major target group for future telemedicine services in Germany, thus it is of particular importance to explore the telemedicine use behavior of this age group. In addition, longitudinal data were exploited, which enabled us to consider two different pandemic stages and account for the exceptional circumstances during that time. Since only few studies observed determinants of online health consultation use in German middle-aged and older adults in the past, our study adds valuable knowledge to the existing literature.

Nevertheless, some limitations should be noted. Telemedicine use was represented by having online consultations with doctors or therapists in our study. We neither examined specific patient groups nor focused on a certain telemedicine format (eg, video conferences or mobile apps). Therefore, we observed a potentially heterogeneous user group. Since telemedicine acceptance during the pandemic was found to vary across medical specialties and telemedicine formats [31], future research that tests for differences in use among patient groups or different telemedicine formats is needed to tailor future services to major user groups. However, our study provides initial insights into telemedicine use in middle and old age. In addition, the DEAS panel holds a slight selection bias. Young, highly educated, healthier, and female individuals were somewhat more likely to participate in the DEAS [83]. However, selection bias in the DEAS sample was found to be small and the distribution of major sociodemographic characteristics closely mirrors the distribution within the overall population of Germany [47]. Moreover, only individuals with access to the internet were included in our study. Therefore, generalization of the results might be slightly limited for some groups of the German middle-aged and older population.

### Conclusion

Telemedicine services represent a valuable tool to deal with the increasing demand for health care caused by population aging. Knowledge about telemedicine use and its determinants, particularly in middle-aged and older individuals, is essential to promote widespread implementations in the future. Our study highlights the relationship of education, psychosocial, and health factors with telemedicine use of community-dwelling middle-aged and older individuals in Germany during the COVID-19 pandemic. Therefore, telemedicine use does not only depend on health needs of middle-aged and older patients. The finding that particularly highly educated individuals used online health consultations may point toward social inequality among telemedicine users. Consequently, efforts should be made to enable access to telemedicine for all patient groups and individual support should be provided (eg, for patients with low [digital] health literacy) to remove barriers to telemedicine use. Moreover, special attention should be paid to individuals with low life satisfaction and an unhealthy lifestyle since they seem harder to reach through telemedicine services. Finally, future research is needed to test the relevance of the observed relationships in the postpandemic context and identify potential reasons for use or nonuse of telemedicine services in middle-aged and older adults in Germany (eg, based on qualitative data). Moreover, cross-country comparisons regarding the determinants of telemedicine use remain to be explored.

## Supplementary material

10.2196/60311 Multimedia Appendix 1 Results of random effects logistic regression for determinants of online health consultation use during the COVID-19 pandemic and additional analyses stratified by sex and age.

## Abbreviations

CES-D: Center of Epidemiologic Studies - Depression scale
DEAS: Deutsches Alterssurvey (German Ageing Survey)
ISCED: International Standard Classification of Education
OR: odds ratio
SHARE: Survey of Health, Ageing and Retirement in Europe
WHO: World Health Organization
