# Supplementary material for: Determinants of Having Online Health Consultations During the COVID-19 Pandemic Among Middle-Aged and Older Adults in Germany: Representative Longitudinal Survey Study
Source: JMIR Aging. 2025 May 26;8:e60311. doi: 10.2196/60311 (PMC12129372; doi:10.2196/60311)
Supplement: Multimedia Appendix 1 [file aging-v8-e60311-s001.docx]

**Table S1.** Results of random effects logistic regression for determinants of online health consultation use during the COVID-19 pandemic.

| **Independent Variables** | **Results of random effects logistic regression** |  |
| --- | --- | --- |
|  | |  |
| Female sex | | 0.83 |
|  | | (0.61 - 1.13) |
| Age | | 1.00 |
|  | | (0.98 - 1.03) |
| High educational level | | 1.43* |
|  | | (1.06 - 1.93) |
| Employment status (ref: employed) | |  |
| Retired | | 0.66+ |
|  | | (0.42 - 1.06) |
| Other/unemployed | | 0.51+ |
|  | | (0.24 - 1.05) |
| Household income | | 1.00 |
|  | | (1.00 - 1.00) |
| Migration background | | 1.31 |
|  | | (0.67 - 2.58) |
| Area lived in (ref: metropolitan districts) | |  |
| Urban districts | | 1.12 |
|  | | (0.79 - 1.61) |
| (Partially) densely populated rural districts | | 0.91 |
|  | | (0.60 - 1.38) |
| Sparsely populated rural districts | | 0.78 |
|  | | (0.49 - 1.27) |
| Residential form of partnership (ref: no partner) | |  |
| Partner in the same household | | 1.34 |
|  | | (0.90 - 2.00) |
| Partner not in the same household | | 1.47 |
|  | | (0.73 - 2.98) |
| Having children | | 1.33 |
|  | | (0.83 - 2.12) |
| Self-rated health | | 0.60*** |
|  | | (0.49 - 0.75) |
| Frequency of physical activity (ref: low frequency) | |  |
| Medium frequency | | 1.58** |
|  | | (1.15 - 2.17) |
| High frequency | | 1.74* |
|  | | (1.09 - 2.76) |
| Frequency of walks (ref: low frequency) | |  |
| Medium frequency | | 1.07 |
|  | | (0.79 - 1.45) |
| High frequency | | 1.33 |
|  | | (0.92 - 1.90) |
| Depressive symptoms | | 1.14 |
|  | | (0.96 - 1.35) |
| Loneliness | | 1.43* |
|  | | (1.06 - 1.93) |
| Life satisfaction | | 1.33* |
|  | | (1.02 - 1.73) |
| Attitute towards own aging | | 1.15 |
|  | | (0.81 - 1.64) |
| Perceiving Corona crisis as a personal threat | | 1.08* |
|  | | (1.01 - 1.15) |
| Oneself infected with Coronavirus (ref: no) | |  |
| Yes | | 1.49 |
|  | | (0.40 - 5.55) |
| Unknown | | 1.06 |
|  | (0.23 - 4.83) |  |
| People from personal environment infected with Coronavirus (ref: no) |  |  |
| Yes | | 0.74+ |
|  | | (0.53 - 1.04) |
| Unknown | | 1.16 |
|  | | (0.41 - 3.26) |
| Feeling that one can influence the infection with Coronavirus | | 1.00 |
|  | | (0.92 - 1.10) |
| Constant | | 0.00*** |
|  | | (0.00 - 0.04) |
|  | |  |
| Observations | | 5,456 |
| Individuals | | 3,222 |

*Note.* Odds Ratios are reported with 95% confidence intervals in parentheses. Unless stated otherwise, the reference category is always zero/absence of the characteristic. ref = reference category. *** p<.001, ** p<.01,* p<.05, + p<.10.

**Table S2.** Results of random effects logistic regression for determinants of online health consultation use during the COVID-19 pandemic stratified by sex.

| **Independent Variables** | **Male** | **Female** |
| --- | --- | --- |
|  |  |  |
| Age | 1.00 | 1.01 |
|  | (0.97 - 1.03) | (0.97 - 1.04) |
| High educational level | 1.70* | 1.21 |
|  | (1.12 - 2.56) | (0.77 - 1.91) |
| Employment status (ref: employed) |  |  |
| Retired | 0.86 | 0.53+ |
|  | (0.45 - 1.62) | (0.26 - 1.06) |
| Other/unemployed | 0.28+ | 0.66 |
|  | (0.07 - 1.07) | (0.26 - 1.67) |
| Household income | 1.00 | 1.00 |
|  | (1.00 - 1.00) | (1.00 - 1.00) |
| Migration background | 0.94 | 1.90 |
|  | (0.37 - 2.38) | (0.69 - 5.24) |
| Area lived in (ref: metropolitan districts) |  |  |
| Urban districts | 1.14 | 1.11 |
|  | (0.71 - 1.84) | (0.64 - 1.93) |
| (Partially) densely populated rural districts | 0.93 | 0.90 |
|  | (0.53 - 1.65) | (0.48 - 1.71) |
| Sparsely populated rural districts | 0.64 | 0.97 |
|  | (0.33 - 1.22) | (0.47 - 2.03) |
| Residential form of partnership (ref. no partner) |  |  |
| Partner in the same household | 2.27* | 0.96 |
|  | (1.13 - 4.59) | (0.57 - 1.62) |
| Partner not in the same household | 2.86* | 0.81 |
|  | (1.04 - 7.87) | (0.27 - 2.47) |
| Having children | 1.38 | 1.11 |
|  | (0.71 - 2.69) | (0.56 - 2.21) |
| Self-rated health | 0.59*** | 0.62** |
|  | (0.44 - 0.79) | (0.45 - 0.85) |
| Frequency of physical activity (ref: low frequency) |  |  |
| Medium frequency | 1.88** | 1.22 |
|  | (1.22 - 2.90) | (0.75 - 1.96) |
| High frequency | 2.17* | 1.32 |
|  | (1.17 - 4.03) | (0.65 - 2.70) |
| Frequency of walks (ref: low frequency) |  |  |
| Medium frequency | 1.02 | 1.13 |
|  | (0.68 - 1.53) | (0.72 - 1.79) |
| High frequency | 1.99** | 0.81 |
|  | (1.24 - 3.20) | (0.45 - 1.44) |
| Depressive symptoms | 1.12 | 1.16 |
|  | (0.88 - 1.42) | (0.91 - 1.49) |
| Loneliness | 1.94** | 1.05 |
|  | (1.25 - 2.99) | (0.68 - 1.62) |
| Life satisfaction | 1.30 | 1.44+ |
|  | (0.91 - 1.85) | (0.97 - 2.15) |
| Attitute towards own aging | 1.48 | 0.81 |
|  | (0.92 - 2.36) | (0.46 - 1.40) |
| Perceiving Corona crisis as a personal threat | 1.08+ | 1.09+ |
|  | (0.99 - 1.18) | (0.99 - 1.20) |
| Oneself infected with Coronavirus (ref: no) |  |  |
| Yes | 1.39 | 1.49 |
|  | (0.25 - 7.74) | (0.19 - 11.71) |
| Unknown | 1.19 | 0.76 |
|  | (0.17 - 8.26) | (0.07 - 8.80) |
| People from personal environment infected with Coronavirus (ref: no) |  |  |
| Yes | 0.68 | 0.78 |
|  | (0.42 - 1.09) | (0.47 - 1.28) |
| Unknown | 0.99 | 1.90 |
|  | (0.27 - 3.61) | (0.34 - 10.80) |
| Feeling that one can influence the infection with Coronavirus | 0.98 | 1.02 |
|  | (0.87 - 1.11) | (0.89 - 1.18) |
| Constant | 0.00*** | 0.01* |
|  | (0.00 - 0.02) | (0.00 - 0.69) |
|  |  |  |
| Observations | 2,783 | 2,673 |
| Individuals | 1,636 | 1,586 |

*Note.* Odds Ratios are reported with 95% confidence intervals in parentheses. Unless stated otherwise, the reference category is always zero/absence of the characteristic. ref = reference category. *** p<.001, ** p<.01,* p<.05, + p<.10.

**Table S3.** Results of random effects logistic regression for determinants of online health consultation use during the COVID-19 pandemic stratified by age groups.

| **Characteristics** | **≤64 Years** | **≥65 Years** |
| --- | --- | --- |
| Female sex | 0.87 | 0.86 |
|  | (0.55 - 1.38) | (0.57 - 1.30) |
| Age | 0.98 | 1.03* |
|  | (0.93 - 1.03) | (1.00 - 1.07) |
| High educational level | 1.26 | 1.58* |
|  | (0.79 - 2.01) | (1.06 - 2.37) |
| Employment status (ref: employed) |  |  |
| Retired | 1.21 | 0.64 |
|  | (0.59 - 2.46) | (0.21 - 1.92) |
| Other/unemployed | 0.65 | 0.36 |
|  | (0.29 - 1.49) | (0.06 - 2.20) |
| Household income | 1.00 | 1.00 |
|  | (1.00 - 1.00) | (1.00 - 1.00) |
| Migration background | 1.52 | 1.28 |
|  | (0.56 - 4.10) | (0.49 - 3.32) |
| Area lived in (ref: metropolitan districts) |  |  |
| Urban districts | 1.26 | 1.06 |
|  | (0.72 - 2.23) | (0.66 - 1.70) |
| (Partially) densely populated rural districts | 1.03 | 0.85 |
|  | (0.53 - 2.00) | (0.49 - 1.48) |
| Sparsely populated rural districts | 0.86 | 0.73 |
|  | (0.40 - 1.84) | (0.38 - 1.37) |
| Residential form of partnership (ref. no partner) |  |  |
| Partner in the same household | 1.01 | 1.66+ |
|  | (0.53 - 1.94) | (0.98 - 2.82) |
| Partner not in the same household | 0.92 | 1.99 |
|  | (0.31 - 2.68) | (0.76 - 5.23) |
| Having children | 1.10 | 1.76 |
|  | (0.58 - 2.10) | (0.86 - 3.61) |
| Self-rated health | 0.59** | 0.61*** |
|  | (0.42 - 0.83) | (0.46 - 0.81) |
| Frequency of physical activity (ref: low frequency) |  |  |
| Medium frequency | 1.91* | 1.52* |
|  | (1.14 - 3.21) | (1.00 - 2.29) |
| High frequency | 2.13+ | 1.60 |
|  | (0.95 - 4.81) | (0.90 - 2.86) |
| Frequency of walks (ref: low frequency) |  |  |
| Medium frequency | 1.19 | 1.01 |
|  | (0.75 - 1.88) | (0.67 - 1.52) |
| High frequency | 0.95 | 1.57+ |
|  | (0.52 - 1.74) | (0.97 - 2.52) |
| Depressive symptoms | 1.13 | 1.11 |
|  | (0.87 - 1.47) | (0.88 - 1.41) |
| Loneliness | 1.45 | 1.52* |
|  | (0.91 - 2.32) | (1.01 - 2.29) |
| Life satisfaction | 1.44+ | 1.29 |
|  | (0.93 - 2.22) | (0.91 - 1.82) |
| Attitute towards own aging | 1.09 | 1.26 |
|  | (0.62 - 1.92) | (0.79 - 2.00) |
| Perceiving Corona crisis as a personal threat | 1.10+ | 1.07 |
|  | (1.00 - 1.21) | (0.98 - 1.17) |
| Oneself infected with Coronavirus (ref: no) |  |  |
| Yes | 1.08 | 3.19 |
|  | (0.22 - 5.22) | (0.23 - 43.82) |
| Unknown | 0.44 | 3.85 |
|  | (0.06 - 3.18) | (0.23 - 63.87) |
| People from personal environment infected with Coronavirus (ref: no) |  |  |
| Yes | 0.70 | 0.81 |
|  | (0.44 - 1.11) | (0.48 - 1.36) |
| Unknown | 0.57 | 2.63 |
|  | (0.12 - 2.74) | (0.62 - 11.09) |
| Feeling that one can influence the infection with Coronavirus | 0.98 | 1.02 |
|  | (0.84 - 1.13) | (0.90 - 1.15) |
| Constant | 0.02+ | 0.00*** |
|  | (0.00 - 1.41) | (0.00 - 0.01) |
|  |  |  |
| Observations | 2,031 | 3,425 |
| Individuals | 1,226 | 2,074 |

*Note.* Odds Ratios are reported with 95% confidence intervals in parentheses. Unless stated otherwise, the reference category is always zero/absence of the characteristic. ref = reference category. *** p<.001, ** p<.01,* p<.05, + p<.10.
